# Effect of Hyperventilation on Periodic Repolarization Dynamics

**DOI:** 10.3389/fphys.2020.542183

**Published:** 2020-09-18

**Authors:** Dominik Schüttler, Lukas von Stülpnagel, Konstantinos D. Rizas, Axel Bauer, Stefan Brunner, Wolfgang Hamm

**Affiliations:** ^1^Medizinische Klinik und Poliklinik I, University Hospital Munich, Ludwig-Maximilians University Munich (LMU), Munich, Germany; ^2^DZHK (German Centre for Cardiovascular Research), Partner Site Munich, Munich Heart Alliance (MHA), Munich, Germany; ^3^Walter Brendel Centre of Experimental Medicine, Ludwig-Maximilians University Munich (LMU), Munich, Germany; ^4^University Hospital for Internal Medicine III, Medical University Innsbruck, Innsbruck, Austria

**Keywords:** hyperventilation, autonomic function, sympathetic nervous system, repolarization instability, T wave vector, periodic repolarization dynamics

## Abstract

Heart and lung functions are closely connected, and the interaction is mediated by the autonomic nervous system. Hyperventilation has been demonstrated to especially activate its sympathetic branch. However, there is still a lack of methods to assess autonomic activity within this cardiorespiratory coupling. Periodic repolarization dynamics (PRD) is an ECG-based biomarker mirroring the effect of efferent cardiac sympathetic activity on the ventricular myocardium. Its calculation is based on beat-to-beat variations of the T wave vector (*dT*°). In the present study, we investigated the effects of a standardized hyperventilation maneuver on changes of PRD and its underlying *dT*° signal in 11 healthy subjects. In response to hyperventilation, *dT*° revealed a characteristic pattern and normalized *dT*° values increased significantly compared to baseline [0.063 (IQR 0.032) vs. 0.376 (IQR 0.093), *p* < 0.001] and recovery [0.082 (IQR 0.029) vs. 0.376 (IQR 0.093), *p* < 0.001]. During recovery, *dT*° remained on a higher level compared to baseline (*p* = 0.019). When calculating PRD, we found significantly increased PRD values after hyperventilation compared to baseline [3.30 (IQR 2.29) deg^2^ vs. 2.76 (IQR 1.43) deg^2^, *p* = 0.018]. Linear regression analysis revealed that the increase in PRD level was independent of heart rate (*p* = 0.63). Our pilot data provide further insights in the effect of hyperventilation on sympathetic activity associated repolarization instability.

## Introduction

Our breathing and heart rate as well as heart function are linked and controlled by the autonomic nervous system. This close connection has been described earlier with heart rates slowing during expiration and a relative tachycardia evolving during inspiration due to vagolytic effects, the so called respiratory sinus arrhythmia (RSA; Yasuma and Hayano, 2004). It is hypothesized that the RSA facilitates efficient respiratory gas exchanges and decreases the workload of the heart while maintaining blood gases in physiological levels (Ben-Tal et al., 2012). Respiratory dysfunction can influence cardiovascular health, and cardiovascular diseases are often associated with respiratory diseases (Garcia et al., 2013). Breathing disorders and pulmonary diseases are tightly linked to autonomic dysfunction (van Gestel and Steier, 2010; Milagro et al., 2019). On the other hand, it has been demonstrated that the maintenance of correct cardiorespiratory coupling exerts beneficial cardiovascular effects in patients with an attenuated RSA (Ben-Tal et al., 2012).

Hyperventilation especially activates the sympathetic nervous system and results in physiological changes of the cardiovascular system: it increases heart rate and blood pressure most likely due to attenuated baroreceptor sensitivity (Alexopoulos et al., 1995; Van De Borne et al., 2000). Concomitant loss of arterial carbon dioxide levels has been connected to various diseases including cerebral and cardiorespiratory disorders (Laffey and Kavanagh, 2002). Additionally, hyperventilation has been demonstrated to affect the repolarization phase of the cardiac cycle by inducing repolarization abnormalities including ST depression and T wave inversion (Alexopoulos et al., 1996).

Respiratory changes exert influences on autonomic nervous activity and thus controlled breathing maneuvers have been used to evaluate autonomic activity and detect dysfunctional states in cardiovascular diseases (Badra et al., 2001). Hawkins et al. (2019), for example, found significantly reduced responses of heart rate in patients with heart failure and coronary heart disease during hyperventilation compared to a healthy cohort.

To non-invasively assess autonomic function, different heart rate and ECG-based biomarkers have been established so far. Here, especially parameters derived from beat-to-beat alterations of the T wave such as microvolt T wave alternans and periodic repolarization dynamics (PRD) have been of increasing interest as especially the repolarization phase is modulated by the sympathetic nervous system and these parameters have been shown to predict the risk for the development of malignant arrhythmias and mortality (Kaufman et al., 2006; Salerno-Uriarte et al., 2007; Verrier et al., 2011; Rizas et al., 2014, 2017; Aro et al., 2016; Bauer et al., 2019). PRD is an ECG-based biomarker which most probably reflects the effect of sympathetic nervous activity on the ventricular myocardium. Its calculation is based on the quantification of low-frequency oscillations (≤0.1 Hz) of cardiac repolarization. In a first step, the angle between successive repolarization vectors (*dT°*) is determined. Subsequently, low-frequency components are assessed using wavelet analysis (Rizas et al., 2014). It is known that increased PRD is associated with increased mortality and cardiovascular mortality in patients with ischemic as well as non-ischemic cardiomyopathy (Rizas et al., 2014, 2017; Bauer et al., 2019).

In this manuscript, we sought to investigate the effect of a standardized hyperventilation maneuver on PRD in a cohort comprised of healthy individuals.

## Materials and Methods

In the present study, we included 11 healthy adults (nine men, two women, and mean age 31.0 years in a range of 25–49 years). We performed a standardized hyperventilation maneuver that has been described in different studies before (Guensch et al., 2014; Fischer et al., 2016; Roubille et al., 2017): in brief, after a resting phase in a seated position for 10 min, volunteers performed hyperventilation at a respiratory rate of 30/min for 1 min followed by an apnea phase as long as tolerated. Afterward, study participants stayed in seated rest until the end of the study. During the entire study time of 20 min, we tracked the spatiotemporal properties of cardiac repolarization on a beat-to-beat basis *via* a high-resolution ECG (Schiller medilog AR4 plus, 1,000 Hz) in orthogonal Frank-lead configuration. Standardized ECG filter settings (high-pass 0.1 Hz; low-pass 100 Hz) were used. The ECG-signals were analyzed using MATLAB with established algorithms for calculation of PRD. In particular, for the assessment of *dT*°, the spatiotemporal information of each T wave has been firstly integrated into a single vector *T*°. The instantaneous degree of repolarization instability was subsequently calculated by means of the angle *dT*° between two successive *T*° vectors and plotted over time (Rizas et al., 2014). PRD is calculated by the use of wavelet analysis in the low-frequency spectrum (≤0.1 Hz). PRD was calculated out of 5 min ECG intervals: PRD before hyperventilation (baseline) was computed between 5 and 10 min. PRD after hyperventilation was calculated within the 5-min interval directly following the apnea phase of each volunteer. The PRD calculation was performed as previously described (Rizas et al., 2014).

Normalization of the *dT*° signal and illustration of data were performed using R-software. Additionally, we recorded high-resolution ECGs (1,000 Hz) in Frank-lead configuration during spirometry-controlled ventilation at breathing rates of 10 and 20/min with constant and normal minute ventilation (tidal volume: 6–8 ml/kg; estimated dead space for adjustment at different breathing rates: 2 ml/kg) in 10 volunteers to check influences of breathing rates on *dT*° and PRD levels. Furthermore, we performed an isometric handgrip (IHG) test in 10 individuals. The maximal voluntary contraction of the dominant forearm was estimated from three 3-s attempts by the use of a digital dynamometer (Takei Digital Hand Grip Dynamometer). After a 5-min baseline period, subjects had to perform a 2-min period of IHG at 30% of maximal voluntary contraction using their dominant forearm. This was followed by a 5-min recovery period. Throughout the test, we continuously recorded high-resolution ECGs (1,000 Hz) in Frank-lead configuration and calculated PRD from the 5-min segments before and directly after the isometric exercise.

Values show median and interquartile ranges. Mann-Whitney-Wilcoxon test was used to reveal statistical differences between *dT*° values before, during, and after hyperventilation as well as PRD values before and after hyperventilation. Significance was indicated by two-sided values of *p* < 0.05. Spearman analyses were used to detect the correlation between *dT*° and heart rate as well as PRD and heart rate before and after hyperventilation. Linear regression analysis was performed to test the association between PRD and change in heart rate (dependent variable: PRD after hyperventilation; independent variables: PRD at baseline before hyperventilation and differences in heart rate before and after hyperventilation).

## Results

**Figure 1:**
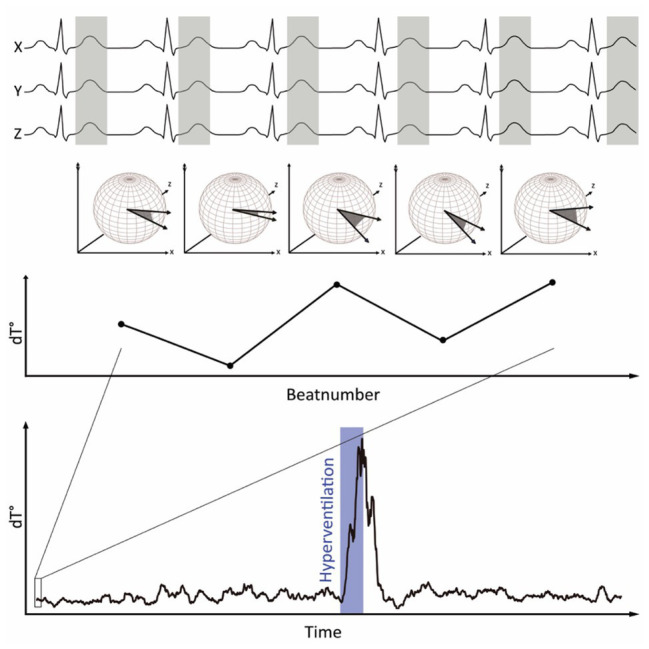
Principle of *dT*° calculation: spatiotemporal information of each T wave is integrated into a single vector *T*°. The instantaneous degree of repolarization instability is calculated by the angle *dT*° between two successive *T*° vectors and plotted over time. During hyperventilation, *dT*° shows a characteristic pattern.

**Figure 2:**
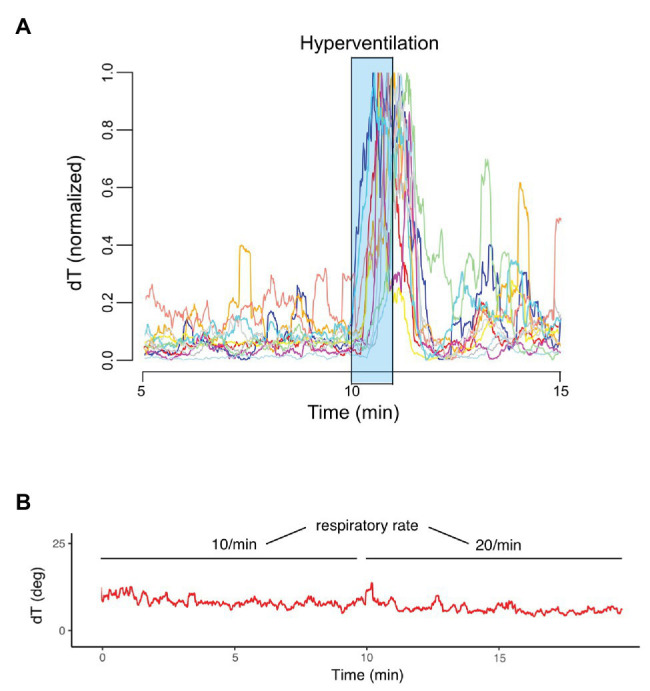
Normalized *dT*° signals of each study participant (*n* = 11). **(A)** *dT*° signal at two different respiratory rates (10 and 20/min) and normal minute ventilation **(B)**.

Figure 1 visualizes the principle of *dT*° assessment from orthogonal ECG-leads. *dT*° signals showed a characteristic pattern in response to hyperventilation: after start of the breathing maneuver, *dT*° signals increased markedly with a noticeable delay. *dT*° signals peaked at the end or slightly after termination of hyperventilation. Figure 1 (lower panel) shows a schematic illustration of the *dT*° signal during our experimental setting. Figure 2A shows normalized *dT*° data for all 11 study participants. During hyperventilation, normalized *dT*° signals significantly increased compared to baseline [0.376 (IQR 0.093) vs. 0.063 (IQR 0.032), *p* < 0.001] and recovery [0.376 (IQR 0.093) vs. 0.082 (IQR 0.029), *p* < 0.001].

During recovery, *dT*° signals remained significantly elevated compared to levels detected in the resting phase before hyperventilation (*p* = 0.019). Mean heart rate (MHR) changed concordantly to the increased *dT*° signals: MHR increased significantly during hyperventilation [91.1 (IQR 10.2) bpm] compared to resting phase [73.2 (IQR 5.7) bpm; *p* < 0.001] and decreased in the recovery phase [72.6 (IQR 7.2) bpm; *p* < 0.001 for hyperventilation vs. recovery]. The recorded *dT*° signal was further used to assess PRD in order to investigate sympathetic activity associated repolarization instability.

**Figure 3:**
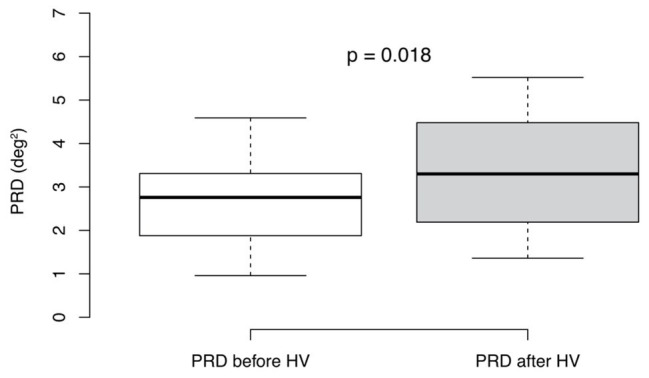
Periodic repolarization dynamics (PRD) levels before and after hyperventilation (HV). Mann-Whitney-Wilcoxon test (*p* < 0.05 estimated as statistically significant), *n* = 11. Boxplots show medians + interquartile ranges.

We detected significantly increased values of PRD after hyperventilation compared to baseline levels [3.30 (IQR 2.29) deg^2^ vs. 2.76 (IQR 1.43) deg^2^; *p* = 0.018; Figure 3]. Moreover, there was no association between the difference of mean *dT*° and the differences of MHRs (*R* = 0.17, *p* = 0.61; Spearman correlation) as well as between the difference of mean PRD and the difference of MHRs before and after hyperventilation (*R* = −0.032; *p* = 0.93; Spearman correlation). Furthermore, we found no correlation between absolute levels of *dT*° and heart rate before (*R* = −0.33, *p* = 0.33; Spearman correlation) or after hyperventilation (*R* = −0.23, *p* = 0.5; Spearman correlation). Similar results were found for absolute levels of PRD and heart rate before (*R* = 0.036, *p* = 0.92; Spearman correlation) and after hyperventilation (*R* = 0.39, *p* = 0.24; Spearman correlation).

Of note, *dT*° signals remained stable when comparing respiratory rates of 10 and 20/min with constant minute ventilation in an additional experiment [7.16 (IQR 1.749) vs. 6.86 (IQR 2.50), *p* = 0.32]. Figure 2B shows an exemplary *dT*° signal of one study participant. Also, PRD levels during controlled breathing (10 vs. 20/min) with constant minute ventilation revealed no statistically significant difference [PRD_10/min_ = 3.17 (IQR 0.30) vs. PRD_20/min_ = 3.08 (IQR 0.36), *p* = 0.275, *n* = 10].

To further exclude heart rate changes as a confounding factor, we performed linear regression analysis. The level of PRD after hyperventilation was not associated with baseline levels of PRD and change of heart rate before and after hyperventilation (*p* = 0.63).

Isometric handgrip tests at 30% of maximal voluntary contraction showed no significant difference between PRD levels before (2.82, IQR 1.74) and after (3.17, IQR 1.77) contraction (*p* = 0.541).

## Discussion

In the present study, we were able to detect a characteristic repolarization pattern in a cohort of healthy young adults during a standardized hyperventilation test. PRD levels were significantly higher after the breathing maneuver. Hyperventilation thus seems to represent a non-invasive method to induce efferent sympathetic activity on the ventricular myocardium.

T wave signals can be affected by breathing patterns *per se* due to a shift in electrical axis. Nevertheless, we saw increased *dT*° signals with a delay after the onset of hyperventilation with the peak of *dT*° close to the end of hyperventilation. Additionally, spirometry-controlled changes of breathing rates with constant minute ventilation had no influence on *dT*° and PRD levels. Moreover, PRD explicitly quantifies low-frequency patterns of repolarization components (≤0.1 Hz). High-frequency components, as those observed because of electrical axis shift during hyperventilation are actively filtered out during calculation of PRD. This effect has been shown using cross-spectral analysis between PRD and respiratory rate in a swine model (Rizas et al., 2014).

In the present study, there was no correlation between heart rate changes and *dT*° as well as PRD changes. Previous studies showed that an increase of heart rate by means of fixed atrial pacing had no relevant effect on *dT*° and PRD levels (Rizas et al., 2014; Hamm et al., 2019). Furthermore, linear regression analysis excluded heart rate as a confounding factor for increased sympathetic activity associated repolarization instability. Nevertheless, efferent cardiac sympathetic activity remained elevated when calculating PRD after hyperventilation compared to baseline levels. Our findings are in line with a recent study, where increased muscle sympathetic nerve activity (MSNA) could be measured during the apnea phase following a short phase of hyperventilation (Eckberg et al., 2016). We additionally performed an IHG test as this maneuver has been demonstrated to increase sympathetic activity (Ray and Carrasco, 2000). Here, we were not able to detect significant changes of PRD levels, indicating no influence on sympathetic activity mediated repolarization instability. Activation of the sympathetic nervous system *via* IHG has been demonstrated by the use of microelectrodes into the peroneal nerve (Victor et al., 1988; Ray and Carrasco, 2000) or pupil dilation responses (Nielsen and Mather, 2015). However, it should be noted, that measurements of sympathetic arousal of the skeletal muscle might not be representative of efferent cardiac sympathetic activity. In our experiments, we were not able to detect changed PRD levels after IHG but after hyperventilation indicating differences between those two provocation tests regarding sympathetic activation.

Our pilot data provide further insights in the effect of hyperventilation on sympathetic activity associated repolarization instability. This simple and easily reproducible test could be useful to detect underlying autonomic dysfunctions in patients with cardiovascular or pulmonary/breathing disorders such as heart failure or sleep apnea.

## Data Availability Statement

The datasets generated for this study are available on request to the corresponding author.

## Ethics Statement

The studies involving human participants were reviewed and approved by Ethikkommission der Medizinischen Fakultät der LMU München. The patients/participants provided their written informed consent to participate in this study.

## Author Contributions

DS performed the experiments and prepared the manuscript. LS, KR, and AB revised the manuscript. SB had the idea for the study and was responsible for conducting the study. WH performed the experiments, designed the figure, and performed statistical analyzes. All authors contributed to the article and approved the submitted version.

### Conflict of Interest

The authors declare that the research was conducted in the absence of any commercial or financial relationships that could be construed as a potential conflict of interest.
